# Investigação Citogenômica de Crianças com Doença Cardíaca Congênita: Experiência de um Centro no Brasil

**DOI:** 10.36660/abc.20190894

**Published:** 2021-11-17

**Authors:** Marcília Sierro Grassi, Marília Montenegro, Evelin Aline Zanardo, Antonio Carlos Pastorino, Mayra Barros Dorna, Chong Kim, Marcelo Jatene, Nana Miura, Leslie Kulikowski, Magda Carneiro-Sampaio

**Affiliations:** 1 Universidade de São Paulo Hospital das Clínicas São Paulo SP Brasil Universidade de São Paulo Hospital das Clínicas , São Paulo , SP - Brasil; 2 Universidade de São Paulo São Paulo SP Brasil Universidade de São Paulo , São Paulo , SP - Brasil

**Keywords:** Cardiopatias Congênitas, Anormalidades Congênitas, Diagnóstico Precoce, Síndrome de DiGeorge, Deleção Cromossômica, Recém-Nascido

## Abstract

**Fundamento:**

Algumas síndromes têm características específicas e facilmente reconhecíveis, enquanto outras podem ser mais complexas de se identificar e podem apresentar diferentes manifestações fenotípicas, por exemplo. Um diagnóstico etiológico é importante para entender a natureza da doença, para estabelecer o prognóstico e para começar o tratamento, permitindo a inclusão de pacientes na sociedade e reduzindo o custo financeiro dessas doenças.

**Objetivo:**

A proposta inicial deste estudo foi a triagem citogenética para detectar a síndrome de deleção 22q11.2 (SD22q11.2) em recém-nascidos e crianças com doença cardíaca congênita utilizando a técnica da amplificação multiplex de sondas dependente de ligação (MLPA). Assim, por meio da pesquisa, outras mudanças genômicas foram identificadas nesses pacientes cardíacos. Nosso objetivo se estendeu a investigar essas outras mudanças citogenéticas.

**Métodos:**

Investigamos 118 recém-nascidos com doenças cardíacas congênitas nascidos consecutivamente durante um ano, utilizando a técnica da MLPA.

**Resultados:**

A técnica da MLPA permitiu a detecção da SD22q11.2 em 10/118 pacientes (8,5%). Outras alterações genômicas foram identificadas em 6/118 pacientes (5%): 1p36 del, 8p23 del (2 casos), 7q dup, 12 dup e 8q24 dup.

**Conclusão:**

Este estudo ressalta a relevância da detecção de alterações genômicas que estão presentes em recém-nascidos e crianças com doenças cardíacas congênitas por meio de ferramentas citogenômicas.

## Introdução

As anomalias congênitas são detectadas em aproximadamente 6% dos recém-nascidos – a literatura internacional descreve que 7,9 milhões de crianças nascem com doenças congênitas graves por ano. No Brasil, as malformações representam a segunda principal causa de mortalidade infantil. ^1^

A doença cardíaca congênita (DCC), de acordo com a definição proposta por Mitchell et al., consiste de uma anomalia estrutural macroscópica do coração ou dos grandes vasos intratorácicos com repercussões funcionais significativas ou potencialmente significativas. Representa aproximadamente 40% de todas as malformações congênitas. De acordo com a Organização Mundial da Saúde (OMS), sua incidência varia de 0,8% em países de alta renda a 1,2% em países de baixa renda. A incidência média de 1% normalmente é aceita no Brasil e em outros países da América Latina. ^2^

O Brasil registra 2,8 milhões de nascidos vivo por ano, e estima-se que quase 29.000 novos casos de DCC ocorrem a cada ano. ^2^ Aproximadamente 20 a 30% dos recém-nascidos (RN) com doenças cardíacas morrem no período neonatal. DCCs são as malformações com o maior impacto na morbidade e mortalidade infantis, assim como nos custos com saúde pública. ^2 , 4 , 5^

Múltiplos fatores ambientais e genéticos, como mutações, alterações cromossômicas e alterações genéticas, estão inter-relacionados e são a base das DCCs. Considerando a variedade de genes que coordenam o desenvolvimento do coração e os vários mecanismos que simultaneamente guiam o desenvolvimento geral do embrião, é razoável imaginar que as mudanças que interferem nesta organização complexa podem levar a várias malformações. Do ponto de vista genético, é importante avaliar se a DCC é uma ocorrência isolada ou se está associada a outras características, de forma a constituir uma síndrome. ^6^ A SD22q11.2 é considerada como a segunda mudança cromossômica mais associada às DCCs, com incidência de 1:4000-5000 nascidos vivos, e aproximadamente 5% dos pacientes com doenças cardíacas apresentam esta deleção. ^7 - 9^ Anomalias conotruncais, principalmente a tetralogia de Fallot, atresia pulmonar com defeito do septo ventricular, transposição das grandes artérias, truncus arteriosus tipo 1 e arco aórtico interrompido (IAA) tipo B são as DCCs mais associadas à SD22q11.2. ^8 - 10^

É importante notar que outras deleções e/ou duplicações que podem ser encontradas em pacientes cardíacos e sua detecção permitem o acompanhamento multidisciplinar adequado, a avaliação do prognóstico e o aconselhamento genético.

## Pacientes e métodos

### Pacientes

Investigamos 118 recém-nascidos com DCC (69 meninos; idade média de 31,7 dias) hospitalizados para correção cirúrgica de maio de 2012 a junho de 2014. Foram incluídos consecutivamente da Unidade de Cardiologia Pediátrica do Instituto do Coração, no Hospital das Clínicas, Faculdade de Medicina da Universidade de São Paulo (InCor – HCFMUSP), do Centro Neonatal do Instituto da Criança, Hospital das Clínicas, Faculdade de Medicina da Universidade de São Paulo (ICr – HCFMUSP), do Centro de Tratamento Intensivo Neonatal (CTIN2) do ICr-HCFMUSP.

O Comitê de Ética do HCFMUSP aprovou o estudo e todas as famílias dos pacientes assinaram um termo de consentimento.

### Extração de DNA

O DNA genômico foi isolado de 3 mL de sangue periférico, utilizando um kit de isolamento de DNA disponível comercialmente (QIAamp DNA Blood Mini Kit ^®^ , Qiagen, Hilden, Alemanha), de acordo com as instruções do fabricante. A qualidade e a quantidade das amostras de DNA foram determinadas utilizando um fluorímetro Qubit ^®^ 2.0 (Invitrogen, Carlsbad, Califórnia, EUA), e sua integridade foi verificada por meio da eletroforese em gel de agarose.

### Estudos da MLPA

A técnica da amplificação multiplex de sondas dependente de ligação (MLPA) foi realizada com cinco kits diferentes: SALSA P064 para as síndromes de microdeleção e microduplicação mais comuns; P036 e P070 para desequilíbrio subtelomérico; e P356 e P250, específicos para o cromossomo 22q e DiGeorge e Velocardiofacial (MRC-Holland, Amsterdã, Holanda). As reações da MLPA foram realizadas de acordo com o protocolo do fabricante. Analisamos os resultados com o software GeneMarker® (Softgenetics, LLC, State College, Pa., EUA). Os resultados foram considerados anormais quando foram observados picos relativos, com tamanho menor que 0,75 (deleção) ou maior que 1,25 (amostras duplicadas em relação ao normal).

## Resultados

A doença cardíaca congênita mais frequente foi a transposição das grandes artérias, observada em 11,9% dos 118 recém-nascidos, seguida da coarctação da aorta, em 10,2%; tetralogia de Fallot e comunicação intraventricular, em 9,3%; e estenose pulmonar, em 7,6%. Todos os defeitos cardíacos congênitos estão descritos na Tabela 1 . Além da DCC, três desses pacientes tinham hipocalcemia, infecções recorrentes e dismorfismo facial.

**Tabela 1:** – Defeitos cardíacos congênitos presentes nos 118 recém-nascidos incluídos

Defeitos cardíacos congênitos	N	%
**Átrio e grandes veias** • Drenagem venosa pulmonar anômala parcial • Drenagem venosa pulmonar anômala total • Isomerismo atrial direito • Defeito do septo atrial	3 3 3 3	2,6 2,6 2,6 2,6
**Conexão atrioventricular** • Atresia mitral • Atresia tricúspide	2 2	1,7 1,7
**Conexão ventriculoarterial** • Transposição das grandes artérias • Dupla via de saída do ventrículo direito • Dupla via de saída do ventrículo esquerdo • Truncus arteriosus • Dupla via de entrada do ventrículo esquerdo	14 8 4 4 1	11,9 6,8 3,4 3,4 0,8
**Ventrículo** • Coração esquerdo hipoplásico	5	4,2
**Septo ventricular** • Comunicação intraventricular	11	9,3
**Fluxo no trato de saída do ventrículo direito** • Estenose pulmonar valvar • Atresia pulmonar • Estenose pulmonar	9 6 3	7,6 5,2 2,6
**Tetralogia de Fallot e variantes** • Tetralogy de Fallot • Atresia pulmonar com comunicação interventricular	11 4	9,3 3,4
**Fluxo no trato de saída do ventrículo esquerdo** • Estenose aórtica valvar	1	0,8
**Artéria coronária** • Origem anômala da artéria coronária esquerda	1	0,8
**Conexões ventriculoarteriais** Coarctação da aorta Arco aórtico interrompido tipo B Estenose pulmonar	12 4 1	10,2 3,4 0,8

Uma análise molecular pela MLPA detectou deleções típicas na 22q11.2 em 10 dos 118 recém-nascidos (8,5%), sendo sete meninos e três meninas com idade de um a 330 dias. Todos os pacientes apresentaram manifestações típicas da doença. A Tabela 2 mostra os dados clínicos e os kits de MLPA que detectaram a 22q11.2.

**Tabela 2:** – Dados clínicos e análise molecular de 10 pacientes com deleção 22q11.2 típica

Paciente N.	Gênero	Idade no diagnóstico (meses)	*Kits MLPA*
11	M	6,7	P064, P250,P356
47	M	11	P064, P250, P356
53	M	0,1	P064, P250
67	M	0,5	P064, P250, P036
78	F	4	P064, P250, P356
85	M	1,1	P064, P250
103	M	0,4	P064
106	M	1	P250, P356
115	M	9	P064, P250, P356
122	F	2,5	P356

*F: feminino; M: masculino.*

Ao pesquisar a deleção 22q11.2 em pacientes com doenças cardíacas, a técnica da MLPA detectou outras microdeleções ou microduplicações em seis outros pacientes. Todos eram do sexo masculino, com idades variando de um a 44 dias. Os dados relacionados a esses pacientes com outras microduplicações ou microdeleções e os kits MLPA utilizados para o diagnóstico estão demonstrados na Tabela 3 .

**Tabela 3:** – Dados clínicos e análise molecular de seis pacientes com outras alterações genômicas

Paciente N.	Gênero	Idade (dias)	Alteração genômica	Região cromossômica	*Kits MLPA*
1	M	4	Deleção	8p23	P250
8	M	7	Duplicação	7q11.2	P036, P070
15	M	13	Deleção	1p36	P064, P070
34	M	44	Duplicação	12p	P036
51	M	1	Duplicação	8q24	P070
61	M	1	Deleção	8p23	P250

*M: Masculino; D: Dias; M: Meses.

A deleção 8p23 foi confirmada pelo kit 250, que é específico para a região crítica da síndrome de deleção 22q11.2, mas também permite o diagnóstico molecular em outros locais genômicos, como 4q35.2, 8p23.1, 9q34. 3, 10p12.31, 10p14, 10p15.1 e 17p13.3.

Detectamos alterações subteloméricas utilizando os kits P036 e P070 em um caso, e o paciente apresentou a duplicação 7q. Houve mudanças em somente um dos kits subteloméricos. Uma análise molecular mostrou que, em um deles, o kit P036 encontrou a duplicação de 12p, e no outro, o kit P070 encontrou a duplicação de 8q24.

Entre os casos deste estudo, o kit P064 permitiu a identificação de mudanças em outras regiões suscetíveis a rearranjos, como a microdeleção do 1p36.

A triagem MLPA em crianças com DCC levou à detecção precoce do defeito genético em 16 recém-nascidos; dez com deleção 22q11.2 e seis outros (4,2%), conforme segue: deleção 8p23 em dois recém-nascidos; duplicação 8q24 em um recém-nascido; duplicação 7q, duplicação 12p e deleção 1p36.

## Discussão

É importante enfatizar que o diagnóstico precoce da DCC tem crescido devido ao aprimoramento do diagnóstico pré-natal. A literatura internacional mostra que 43,6% dos pacientes cardíacos são diagnosticados na primeira semana de vida; 70%, até seis meses de idade; e até 86% em no máximo dois anos. ^11 - 13^

Como as doenças cromossômicas são mais frequentes em pacientes com malformações cardíacas do que na população em geral, é essencial enfatizar a importância da investigação genética no diagnóstico da DCC. O diagnóstico molecular específico de uma síndrome genética vai permitir um acompanhamento multidisciplinar adequado, uma avaliação prognóstica e a conscientização da família sobre os riscos da recorrência. ^14^

As síndromes da microdeleção e/ou da microduplicação associadas à doença cardíaca congênita não são detectadas no exame clássico do cariótipo; elas podem ser somente identificadas quando utilizamos outras técnicas moleculares, como hibridação *in situ* por fluorescência (FISH), MLPA ou, mais recentemente, análise cromossômica por *microarray* (CMA). ^11^

Este estudo demonstrou a importância de se utilizar a técnica da MLPA para o diagnóstico precoce da SD22q11.2 em pacientes com DCCs no primeiro ano de vida. Observamos que esta deleção foi detectada em 8,5% dos pacientes, o que excedeu as taxas descritas na literatura. Este achado pode estar relacionado às características dos pacientes incluídos no estudo, já que a maioria recebia tratamento em um hospital terciário nacional de referência. ^9 , 10 , 16^

Apesar dos avanços recentes e do fato de a SD22q11.2 ser considerada como a microdeleção cromossômica mais comum em humanos, de acordo com estudos realizados na Europa e nos Estados Unidos, ^8^ esta síndrome permanece subdiagnosticada no Brasil devido à falta de informação entre médicos e à dificuldade de se obter uma triagem específica no Sistema Único de Saúde (SUS). No Brasil, não há estudos sobre a triagem da deleção 22q11.2 utilizando a técnica da MLPA exclusivamente na faixa etária que nós analisamos. Neste período da vida, o fenótipo nem sempre é claro, o que dificulta o diagnóstico precoce nesses pacientes. ^8 , 9 , 12^

Nosso objetivo é enfatizar que as doenças cardíacas congênitas devem ser um sinal de alerta para a SD22q11.2, especialmente em pacientes com malformações conotruncais, hipocalcemia e dismorfismo facial. Porém, ainda é um grande desafio devido aos seus vários aspectos clínicos e comportamentais, que podem atrasar o diagnóstico e o tratamento adequado. Além disso, a importância de se detectar outras deleções e/ou duplicações relacionadas à DCC é clara, já que isso levaria ao aconselhamento genético, ao acompanhamento multidisciplinar e à identificação de outras malformações associadas. ^10 , 12 , 16 , 22^

Os resultados descritos aqui estão alinhados com a literatura, que confirma a presença das malformações conotruncais na maioria dos pacientes com deleção 22q11.2. ^9 , 16^

Pacientes com SD 22q11.2 e DCC severa provavelmente morrem antes de ter acesso ao hospital terciário de referência, levando à subnotificação da sua taxa de ocorrência. É importante notar que, no Brasil, diferentemente de países desenvolvidos, o aborto em casos de malformação não é permitido por lei. Isso também justificaria a diferença quando comparamos nossa série de casos com as taxas de detecção de deleção 22q11.2 descritas na literatura. ^9 , 10 , 17 , 19 , 20^

Neonatologistas, cardiologistas pediátricos e cirurgiões cardíacos devem estar cientes das especificidades e do cuidado associados à SD22q11.2. Como se pode observar, pacientes afetados pela condição normalmente apresentam mudanças em vários sistemas do corpo e requerem muitas intervenções clínicas, envolvendo hospitalização ao longo da vida. Deve-se notar que pacientes com SD22q11.2 têm taxas de mortalidade mais altas em comparação a outras síndromes genéticas (como a Síndrome de Down) e casos não-sindrômicos de DCC. ^16 , 23 , 31^

Dada a importância desta síndrome altamente complexa, é essencial fazer uma triagem e detectá-la cedo no primeiro ano de vida, com base em um conjunto de características clínicas e laboratoriais. ^12^ Vale notar que, neste grupo etário, as taxas de morbidade e mortalidade são altas, principalmente devido a infecções recorrentes resultando de aplasia ou hipoplasia tímica em DCC. ^12 , 34 , 35^

Dentre os pacientes avaliados, a deleção 8p23 foi observada em dois deles. As doenças complexas foram dupla via de saída do ventrículo direito e hipoplasia tricúspide no ventrículo direito, ambas diagnosticadas na primeira semana de vida. É importante salientar que esses pacientes não apresentavam nenhuma anomalia clínica típica.

Vários autores sugeriram que a deleção 8p pode ser mais frequente do que o número relativamente baixo de casos descritos na literatura. Os resultados dos estudos da correlação genético-fenótipo de indivíduos com DCC enfatizam a região cromossômica 8p23.1 como a região crítica para a morfogênese cardíaca. A porção proximal da região p23.1 contém o gene da proteína de ligação 4 (GATA4), que codifica um fator de transcrição que tem papel essencial no desenvolvimento dos corações humanos. CCs neste tipo de deleção podem ser explicadas pela haploinsuficiência do GATA4. ^36^

A deleção 8p23.1 pode estar associada a uma ampla gama de anomalias clínicas, incluindo dismorfismo facial, microcefalia, restrição de crescimento intrauterino, doenças psiquiátricas, déficit intelectual e doenças cardíacas, sendo que as mais frequentes são defeitos do septo atrioventricular e do septo atrial. ^36 , 38 , 39^ Há relatos de deleção intersticial e terminal no braço curto do cromossomo 8 (mais frequentemente com rupturas nas regiões 8p21 a 8p23), assim como deleções combinadas com duplicações. A variável status clínico se deve à extensão da deleção ou à localização do ponto de ruptura. ^38^

O déficit intelectual é o achado mais frequente para este tipo de deleção. Aparentemente há uma relação entre o tamanho da região da deleção no cromossomo 8 e o nível de deficiência intelectual, segundo a qual as deleções terminais mais distais à região 23.1 estão associadas ao dano intelectual menor. ^36 , 40^

Também foi possível observar déficit motor, atraso linguístico e mudanças comportamentais (hiperatividade, déficit de atenção e agressividade). As deleções SOX7 também têm um papel no atraso do desenvolvimento e, possivelmente, nas características dismórficas de indivíduos com deleção 8p23. ^38 , 41 , 42^

Este estudo detectou a duplicação 7q11.2 em um paciente. Diferentemente da deleção de 1p36 e 8p23, este tipo de alteração genômica não tem correlação com doenças cardíacas. O paciente incluído no estudo tinha coarctação da aorta, e o diagnóstico foi feito aos sete dias de vida; não havia nenhuma malformação aparente.

É importante notar que esta é uma patologia rara, já que há aproximadamente 54 casos descritos na literatura. Duplicações parciais 7q foram classificadas em quatro grupos de acordo com a região cromossômica afetada. ^43^

Para o paciente detectado com a duplicação 8p24, haverá um acompanhamento neurológico para monitorar o desenvolvimento clínico, já que os genes associados à epilepsia estão localizados na região 8p24, e alterações nesta região podem ser responsáveis por convulsões tônicas, às vezes seguidas de convulsões clônicas generalizadas. Um dos genes envolvidos é o KCNQ3 (canais de potássio dependentes de voltagem, subfamília Q, membro 3). O desenvolvimento costuma ser benigno, com remissão no primeiro ano de vida. É importante observar que neste tipo de alteração genômica não há maior correlação com alguns tipos específicos de doenças cardíacas. O paciente da nossa série tinha atresia pulmonar e nenhum outro achado clínico. ^44^

A outra alteração genômica detectada em um paciente com IAA foi a duplicação 12p. A literatura demonstra que esta alteração genômica está relacionada à síndrome de Pallister-Killian (SPK), pela qual pacientes apresentam alterações craniofaciais típicas, defeitos cardíacos congênitos, atraso de desenvolvimento variável, dano intelectual, convulsões, hipotonia, surdez, lóbulos da orelha grandes, pescoço curto, mãos grandes com dedos pequenos, encurtamento desproporcional de braços e pernas, deficiências de pigmentação da pele, hérnia diafragmática congênita e outras anomalias sistêmicas. A SPK está relacionada à trissomia ou à tetrassomia de genes específicos localizados na região duplicada. O fenótipo SPK normalmente é observado em indivíduos com duplicações completas ou parciais de 12p (trissomia 12p, em vez de tetrassomia 12p) como resultado da duplicação intersticial ou translocação desequilibrada. ^45^

Nesses casos, a correlação fenótipo-genótipo é muito importante para o diagnóstico clínico e as abordagens terapêuticas. Porém, alguns fenótipos parecem estar associados a duplicações de regiões cromossômicas específicas.

Avanços médicos permitiram mais precisão na correlação entre a estrutura do DNA e o fenótipo clínico. Como demonstrado neste estudo, as técnicas citogenéticas permitem o diagnóstico precoce de diferentes síndromes de deleção e duplicação em pacientes cardíacos. Deve-se notar que um conhecimento aprofundado de testes citogenéticos e sua aplicação em diferentes campos da medicina levarão à escolha adequada para a triagem: MLPA, *array* , painel ou sequenciamento de exoma.

Estudos recomendam que todos os recém-nascidos ou crianças com doenças cardíacas e dismorfismo, ou outras anomalias congênitas, sejam testadas para deleção 22q11.2. Além disso, os desequilíbrios genotípicos de outras regiões cromossômicas, incluindo 10p12-p15, 4q21-q35, 8p21-p23, 17p13 e 18q21, podem ser encontrados em pacientes com suspeita clínica de SD22q11.2 e sem a deleção 22q11.2. ^46 , 47^

A investigação genômica é crucial para realizar um perfil correto e precoce da etiologia de pacientes cardíacos, além de permitir o melhor entendimento da alta variabilidade fenotípica nesses pacientes.

Por fim, acreditamos que este estudo vai ajudar a demonstrar a importância da investigação citogenética precoce em pacientes cardíacos para avaliar o prognóstico e o risco de recorrência, ao mesmo tempo em que leva ao tratamento correto, acompanhamento e aconselhamento familiar.

## Conclusões

Os autores demonstraram a importância de investigar e detectar alterações genômicas utilizando a técnica da MLPA em recém-nascidos e crianças com doenças cardíacas. O rápido progresso do uso de testes citogenéticos no ambiente clínico leva os médicos a gerenciar as DDCs e considerar que muitos pacientes podem sofrer de déficits cromossômicos ou até genéticos.

A detecção de alterações citogenômicas permitirá que médicos avaliem o prognóstico e o risco de recorrência, além de permitir o devido acompanhamento e tratamento, com o objetivo de melhorar a qualidade de vida das crianças.
