# Supplementary material for: Investigação Citogenômica de Crianças com Doença Cardíaca Congênita: Experiência de um Centro no Brasil
Source: Arq Bras Cardiol. 2021 Nov 17;118(1):61–7. [Article in Portuguese] doi: 10.36660/abc.20190894 (PMC8959050; doi:10.36660/abc.20190894)
Supplement: Translation

# Translation: Cytogenomics Investigation of Infants with Congenital Heart Disease: Experience of a Brazilian Center

**Authors:** Marcília Sierro Grassi, Marília Montenegro, Evelin Aline Zanardo, Antonio Carlos Pastorino, Mayra Barros Dorna, Chong Kim, Marcelo Jatene, Nana Miura, Leslie Kulikowski, Magda Carneiro-Sampaio

**Affiliations:** 1 Brasil Universidade de São Paulo Hospital das Clínicas, São Paulo, SP - Brasil; 2 Brasil Universidade de São Paulo, São Paulo, SP - Brasil

**Keywords:** Heart Defects, Congenital, Congenital Abnormalities, Early Diagnosis, DiGeorge Syndrome, Chromosome Deletion, Newborn

## Abstract

**Background:**

Some syndromes have specific and easily recognizable features, while others may be more complex to identify and may present different phenotypic manifestations, for example. An etiological diagnosis is important to understand the nature of the disease, to establish the prognosis and to start the treatment, allowing the inclusion of patients in society and reducing the financial cost of such diseases.

**Objective:**

The initial proposal of this study was cytogenetic screening for the detection of the 22q11.2 deletion syndrome in consecutive newborns and infants with congenital heart disease using the multiplex ligation-dependent probe amplification (MLPA) technique. Therefore, throughout our research, other genomic alterations were identified in these cardiac patients. Thus, our objective was extended to investigate these other cytogenetic alterations.

**Methods:**

We investigated 118 neonates with congenital heart diseases born consecutively during one year using the MLPA technique.

**Results:**

The MLPA technique allowed the detection of 22q11.2DS in 10/118 patients (8.5%). Other genomic alterations were also identified in 6/118 patients (5%): 1p36 del, 8p23 del (2 cases), 7q dup, 12 dup and 8q24 dup.

**Conclusion:**

This study highlights the relevance of detecting genomic alterations that are present in newborns and infants with congenital cardiac diseases using cytogenomic tools.

## Introduction

Congenital anomalies are detected in approximately 6% of newborns – the international literature describes that 7.9 million children are born with severe congenital disorders per year. In Brazil, malformations represent the second-leading cause of child mortality. ^1^

Congenital heart disease, according to the definition proposed by Mitchell et al., consists of a macroscopic structural abnormality of the heart or large intrathoracic vessels, with significant or potentially significant functional repercussions. It represents approximately 40% of all congenital malformations. Its incidence, according to the World Health Organization (WHO), ranges from 0.8% in high-income countries to 1.2% in low-income countries. The average incidence of 1% is usually accepted in Brazil and other Latin American countries. ^2^

Brazil registers 2.8 million live births annually, and it is estimated that almost 29,000 new cases of congenital heart disease (CHD) occur each year ^2^ . Approximately 20% to 30% of newborns (NB) with heart diseases die in the neonatal period. CHDs are the malformations with the greatest impact on infant morbidity and mortality and on public health expenses. ^2 , 4 , 5^

Multiple environmental and genetic factors such as mutations, chromosomal alterations and gene alterations are interrelated and underlie CHDs. Given the variety of genes that coordinate the development of the heart and the various mechanisms that simultaneously guide the overall embryo development, it is reasonable to imagine that changes that interfere with this complex organization can lead to many malformations. From a genetic point of view, it is essential to evaluate whether CHD is an isolated occurrence or if it is associated with other characteristics, in such a way to constitute a syndrome. ^6^ The 22q11.2 deletion syndrome is considered as the chromosomal change that is the second most associated with CHDs, with an incidence of 1:4000-5000 live births, and approximately 5% of the patients with heart diseases present with this deletion. ^7 - 9^ Conotruncal anomalies, particularly tetralogy of Fallot, pulmonary atresia with a ventricular septal defect, transposition of the great arteries, type I truncus arteriosus and type B interrupted aortic arch are the most common CHDs associated to 22q11.2 deletion syndrome. ^8^

It is also important to note that other deletions and/or duplications that may be found in cardiac patients and their detection allow a proper multidisciplinary follow-up, prognostic assessment and genetic counseling.

## Patients and Methods

### Patients

We investigated 118 newborns with congenital heart disease (69 males; mean age of 31.7 days) admitted for surgical correction from March 2012 to June 2014. They were included consecutively from the Pediatric Cardiology Unit of Instituto do Coração, at Hospital das Clinicas, Medical School of Universidade de Sao Paulo (InCor – HCFMUSP), the Neonatal Center of Instituto da Criança, Hospital das Clinicas, Medical School of Universidade de Sao Paulo (ICr – HCFMUSP) or the Intensive Neonatal Treatment Center (CTIN2) of ICr-HCFMUSP.

The ethics committee of HCFMUSP approved the study, and an informed consent form was obtained from the families of all patients.

### DNA extraction

Genomic DNA was isolated from 3 mL of peripheral whole blood using a commercially available DNA isolation kit (QIAamp DNA Blood Mini Kit ^®^ , Qiagen, Hilden, Germany) according to the manufacturer’s instructions. The quality and quantity of DNA samples were determined using a Qubit ^®^ 2.0 Fluorometer (Invitrogen, Carlsbad, California, USA), and the integrity was ascertained by agarose gel electrophoresis.

### MLPA studies

The multiplex ligation-dependent probe amplification (MLPA) technique was performed using five different kits: SALSA P064 for the most common microdeletion and microduplication syndromes, P036 and P070 for subtelomeric imbalance, and P356 and P250, which are specific for chromosome 22q and Digeorge/Velocardial (MRC-Holland, Amsterdam, The Netherlands). The MLPA reactions were performed according to the manufacturer's protocol. We analyzed the results using the software GeneMarker® (Softgenetics, LLC, State College, Pa., USA). The results were considered abnormal when relative peaks were observed, whose size was smaller than 0.75 (deletion) or larger than 1.25 (duplicate samples relative to normal).

## Results

The most frequent congenital heart disease was transposition of the great arteries, observed in 11.9% of 118 neonates, followed by aorta coarctation, in 10.2%; tetralogy of Fallot and interventricular communication, in 9.3%; and pulmonary stenosis in 7.6%. All congenital heart defects are described in Table 1 . In addition to CHD, three of these patients had hypocalcemia, recurrent infections and facial dimorphism.

**Table 1:** – Congenital heart defects present in the 118 newborns included

Congenital heart defects	N	%
**Atrium and large veins** • Partial anomalous pulmonary venous drainage • Total anomalous pulmonary venous drainage • Right atrial isomerism • Atrial septal defect	3 3 3 3	2.6 2.6 2.6 2.6
**Atrioventricular connection** • Mitral atresia • Tricuspid atresia	2 2	1.7 1.7
**Ventriculo-arterial connection** • Transposition of the great arteries • Double-outlet right ventricle • Double-outlet left ventricle • Truncus arteriosus • Double-entry lefty ventricle	14 8 4 4 1	11.9 6.8 3.4 3.4 0.8
**Ventricle** • Hypoplastic left heart	5	4.2
**Ventricular septum** • Interventricular communication	11	9.3
**Right ventricular outflow tract** • Valvar pulmonary stenosis • Pulmonary atresia • Pulmonary stenosis	9 6 3	7.6 5.2 2.6
**Tetralogy of Fallot and variants** • Tetralogy of Fallot • Pulmonary atresia with interventricular communication	11 4	9.3 3.4
**Left ventricular outflow tract** • Valvar aortic stenosis	1	0.8
**Coronary artery** • Anomalous origin of the left coronary artery	1	0.8
**Ventriculo-arterial connections** • Aortic coarctation • Type B interruption of the aortic arch • Pulmonary stenosis	12 4 1	10.2 3.4 0.8

A molecular analysis by MLPA detected typical deletions at 22q11.2 in 10 of 118 neonates (8.5%), being seven males and three females, ranging in age from one to 330 days. All patients presented typical manifestations of the disease. Table 2 shows the clinical data and the MLPA kits that detected the 22q11.2.

**Table 2:** – Clinical data and Molecular analysis of 10 patients with typical 22q11.2 deletion

Patient No.	Gender	Age at diagnosis (months)	*MLPA Kits*
11	M	6.7	P064, P250,P356
47	M	11	P064, P250, P356
53	M	0.1	P064, P250
67	M	0.5	P064, P250, P036
78	F	4	P064, P250, P356
85	M	1.1	P064, P250
103	M	0.4	P064
106	M	1	P250, P356
115	M	9	P064, P250, P356
122	F	2.5	P356

*F: female; M: male.*

When searching for the 22q11.2 deletion in patients with heart disease, the MLPA technique detected other microdeletions or microduplications in six other patients. All of those patients were male, with ages ranging from one day to 44 days. Data regarding those patients with other microduplications or microdeletions and the MLPA kits used for the diagnosis are shown in Table 3 .

**Table 3:** – Clinical data and Molecular analysis of six patients with other genomic alterations

Patient No.	Gender	Age (days)	Genomic Alteration	Chromosome region	*MLPA Kits*
1	M	4	Deletion	8p23	P250
8	M	7	Duplication	7q11.2	P036, P070
15	M	13	Deletion	1p36	P064, P070
34	M	44	Duplication	12p	P036
51	M	1	Duplication	8q24	P070
61	M	1	Deletion	8p23	P250

*M: Male; D: Days; M: months.

The 8p23 deletion was confirmed by kit 250, which is specific for the critical region of the 22q11.2 deletion syndrome, but also allows molecular diagnosis at other genomic loci, such as 4q35.2, 8p23.1, 9q34. 3, 10p12.31, 10p14, 10p15.1 and 17p13.3.

We detected subtelomeric alterations using the P036 and P070 kits in one case, and the patient presented with 7q duplication. There were changes in only one of the subtelomeric kits. A molecular analysis revealed that, in one, the P036 kit found the duplication of 12p, and in the other, the P070 kit found the duplication of 8q24.

Among the cases of this study, the P064 kit allowed the identification of alterations in other regions susceptible to rearrangements, such as a microdeletion in 1p36.

The MLPA screening in infants with CHD led to the early detection of a genetic defect in 16 neonates, ten with 22q11.2 deletion and six other patients (4.2%) as follows: 8p23 deletion in two neonates, 8q24 duplication in one neonate, 7q duplication, 12p duplication and 1p36 deletion.

## Discussion

It is important to highlight that the early diagnosis of CHD has been increasing due to the improvement of prenatal diagnosis. The international literature shows that 43.6% of cardiac patients are diagnosed during the first week of life; 70%, up to 6 months of age; and up to 86% in up to two years. ^11 - 13^

As chromosome disorders are more frequent in patients with cardiac malformations than in the general population, it is crucial to emphasize the importance of genetic investigation in the diagnosis of CHD. The specific molecular diagnosis of a genetic syndrome will allow a proper multidisciplinary follow-up, prognostic assessment and family awareness of the risks of reoccurrence. ^14^

The microdeletion and/or microduplication syndromes associated with congenital heart disease are not detected in classical karyotyping, which may only be identified when we employ other molecular techniques, such as FISH (fluorescence in situ hybridization), MLPA, or, more recently, chromosomal microarray analysis (CMA). ^11^

This study demonstrated the importance of using the MLPA technique for the early diagnosis of 22q11.2DS in patients with CHDs in the first year of life. We observed that this deletion was detected in 8.5% of the patients, exceeding the rates described in the literature. This finding may be owed to the characteristics of the patients included in the study, most of whom were treated at a national tertiary referral hospital. ^9 , 10 , 16^

Despite recent advances and the fact that 22q11.2DS is considered as the most common chromosome microdeletion in humans, according to studies undertaken in Europe and in the United States, ^8^ this syndrome remains underdiagnosed in Brazil due to the lack of information among physicians and the difficulty to obtain a specific screening in the Brazilian Unified Health System (SUS). In Brazil, there are no studies on the screening of the 22q11.2 deletion using the MLPA technique exclusively in the age group that we address. In this period of life, the phenotype is not always clear, which makes early diagnosis very difficult in these patients. ^8 , 9 , 12^

Our purpose is to highlight that congenital heart diseases should be a warning sign for the 22q11.2DS, particularly in patients with conotruncal malformations, hypocalcemia, and facial dysmorphisms. However, it still poses a great challenge due to its many clinical and behavioral features, which can often delay the diagnosis and proper treatment. In addition, the importance of detecting other CHD-related deletions and/or duplications is clear, which would allow genetic counseling, multidisciplinary follow-up and identification of other associated malformations. ^10 , 12 , 16 , 22^

The results described here are in line with the literature, which confirms the presence of conotruncal malformations in most of the patients with 22q11.2 deletion. ^9 , 16^

Patients with the 22q11.2DS and severe CHD probably die before having access to a tertiary referral hospital, leading to the underestimation of the occurrence rate. It is important to note that, in Brazil, unlike in some developed countries, abortion in cases of malformation is not allowed by law. This would also justify the difference when we compare our case series with detection rates of 22q11.2 deletion described in the literature. ^9 , 10 , 17 , 19 , 20^

Neonatologists, pediatric cardiologists and cardiac surgeons should be aware of the specifics and the care associated with 22q11.2DS. As we can see, affected patients often present changes in several body systems and require many clinical interventions involving hospitalization throughout their lives. It should be noted that patients with 22q11.2DS have the highest mortality rates when compared to other genetic syndromes (such as Down syndrome) and nonsyndromic cases of CHD. ^16 , 23 , 31^

Given the importance of this highly complex syndrome, it is crucial to perform a screen and detect it early in the first year of life, based on a set of clinical and laboratory characteristics. ^12^ It is worth noting that, in this age range, morbidity and mortality rates are high, mainly due to recurrent infections resulting from aplasia or thymic hypoplasia and CHD. ^12 , 34 , 35^

Among the assessed patients, the 8p23 deletion was observed in two of them. The complex diseases were infundibular double outlet right ventricle and right ventricular tricuspid hypoplasia, both diagnosed in the first week of life. It is important to highlight that these patients did not have any typical clinical abnormalities.

Several authors have suggested that 8p deletion may be more frequent than the relatively low number of cases described in the literature. The results of genotype-phenotype correlation studies of individuals with CHDs highlighted the chromosome region 8p23.1 as a critical region for cardiac morphogenesis. The proximal portion of the p23.1 band contains the GATA binding protein 4 (GATA4) gene, which encodes a transcription factor that plays a key role in the development of human hearts. CCs in this type of deletion can be explained by the GATA4 haploinsufficiency. ^36^

8p23.1 deletion may be associated with a wide range of clinical abnormalities, including facial dysmorphism, microcephaly, intrauterine growth restriction, neuropsychiatric disorders, intellectual deficit and heart diseases, the most frequent of which are atrioventricular septal defects and atrial septal defects. ^36 , 38 , 39^ There are reports of interstitial and terminal deletions of the short arm of chromosome 8 (most often with breaks in the 8p21 to 8p23 bands), as well as deletions combined with duplications. The variable clinical status is due to the extent of deletion or to the location of the breakpoint region. ^38^

Intellectual deficit is the most frequently described finding for this type of deletion. There appears to be a relationship between the size of the deleted region in chromosome 8 and the degree of intellectual impairment, in which terminal deletions more distal to band 23.1 are associated with lower intellectual impairment. ^36 , 40^

We can also observe motor deficit, language delay and behavioral changes (hyperactivity, attention deficit and aggressiveness). SOX7 deletions have also been understood to have a role in the developmental delay and possibly in the dysmorphic features in individuals with 8p23 deletion. ^38 , 41 , 42^

The present study detected 7q11.2 duplication in one patient. Unlike deletion of 1p36 and 8p23, this type of genomic alteration has no correlation with heart diseases. The patient included in the study had aortic coarctation, and the diagnosis was made at seven days of age, and he did not present with any apparent malformation.

It is important to note that this is a rare pathology, as there are approximately 54 cases described in the literature. Partial 7q duplications were classified into four groups according to the affected chromosome region. ^43^

For the patient detected with 8q24 duplication, there will be a neurological follow-up to monitor the clinical development, since epilepsy-associated genes are located in the 8q24 region, and alterations in this region may be responsible for tonic seizures, sometimes followed by the generalized clonic seizure. One of the genes involved is KCNQ3 (potassium channel, voltage-gated KQT-like subfamily Q member 3). The development is usually benign, with remission in the first year of life. It is important to note that in this type of genomic alteration, there is no greater correlation with some specific types of cardiac diseases. The patient in our series had pulmonary atresia and no other clinical findings. ^44^

The other genomic alteration detected in a patient with IAA was 12p duplication. The literature demonstrates that this genomic alteration is related to Pallister-Killian syndrome (PKS), in which patients present typical craniofacial alterations, congenital heart defects, variable developmental delay, intellectual impairment, seizures, hypotonia, deafness, thick earlobes, short neck, large hands with short fingers, disproportionate shortening of arms and legs, skin pigmentation disorders, congenital diaphragmatic hernia and other systemic abnormalities. PKS is related to the trisomy or tetrasomy of specific genes located in the duplicated region. The PKS phenotype is often observed in individuals with complete or partial duplications of 12p (12p trisomy rather than 12p tetrasomy) as a result of interstitial duplication or unbalanced translocation. ^45^

In these cases, the phenotype-genotype correlation is very important for the clinical, diagnostic and therapeutic approaches. However, certain phenotypes seem to be associated with duplications of particular chromosome regions.

Medical advances have allowed an increasingly accurate correlation between DNA structure and clinical phenotype. As shown in this study, cytogenetic techniques allow the early diagnosis of different deletion and duplication syndromes in cardiac patients. It should be noted that a deep knowledge of cytogenetic tests and their application in different fields of medicine will allow the appropriate choice for the screen: MLPA, array genotyping, panel or exome sequencing.

Studies recommend that all newborns or children with cardiac diseases and dysmorphism or other congenital anomalies be tested for deletion 22q11.2. In addition, genotypic imbalances of other chromosome regions, including 10p12-p15, 4q21-q35, 8p21-p23, 17p13 and 18q21, can be found in patients with clinical suspicion of SD22q11.2 and without 22q11.2 deletion. ^46 , 47^

Genome investigation is crucial to allow an unequivocal and early profile of the etiology of cardiac patients, in addition to allowing a better understanding of the high phenotypic variability in these patients.

Finally, we believe this study will help demonstrate the importance of early cytogenetic investigation in cardiac patients to assess the prognosis and risks of recurrence while allowing proper treatment, follow-up and family counseling.

## Conclusions

The authors demonstrate the importance of investigating and detecting genomic alterations by the MLPA technique present in newborns and infants with cardiac diseases. The fast progress of the use of cytogenetic tests in a clinical environment leads physicians managing CHDs to consider that many patients may suffer from chromosomal or even genetic disorders.

The detection of cytogenomic alterations will allow clinicians to evaluate the prognosis and the risks of recurrence while allowing proper follow-up and treatment, aiming at improving the children’s life quality.
